# A molecular dynamics study of the structure and dynamics of screened polyelectrolyte complex materials

**DOI:** 10.1039/d5sm01081k

**Published:** 2026-01-14

**Authors:** Sophie G. M. van Lange, Nayan Vengallur, Andrea Giuntoli, Jasper van der Gucht

**Affiliations:** a Physical Chemistry and Soft Matter, Wageningen University and Research Stippeneng 4 Wageningen 6708 WE The Netherlands jasper.vandergucht@wur.nl; b Zernike Institute for Advanced Materials, University of Groningen Nijenborgh 3 Groningen 9747 AG The Netherlands a.giuntoli@rug.nl

## Abstract

Compleximers are a novel class of materials that combine the properties of thermoplastics and thermosets, due to their reversible network structure facilitated by ionic interactions moderated by hydrophobic attenuators. In this study, we investigate the structural and dynamic properties of various architectures of these relatively unexplored, water-free polyelectrolyte complex materials using molecular dynamics simulations. We find that decreasing the number of charges per unit mass, either by increasing the length of neutral side chains or by decreasing the charge density of the polymers, leads to a decrease in density. This is related to a decrease in cohesive energy and in the number of contacts between oppositely charged beads. These structural changes result in an enhancement of the dynamics and a lowering of the glass transition temperature (*T*_g_), which correlates with the number of oppositely charged contacts in the first coordination shell of the monomers. The fragility, represented in Angell plots, was found to be universal for all systems, irrespective of charge density. Compleximers also follow the universal correlation between the structural relaxation time and the rattling amplitude in glass-forming liquids.

## Abstract 2

The structural and dynamic properties of compleximers, a novel class of polymer materials crosslinked by attenuated ionic bonds between oppositely charged polymers, are studied using molecular dynamics simulations.

## Graphical abstract

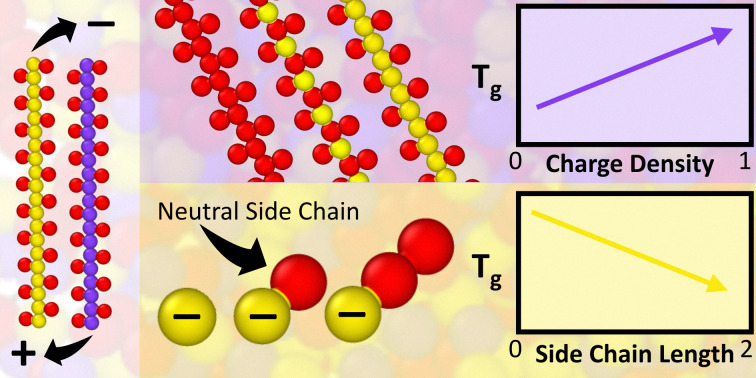

## 1 Introduction

Thermosetting polymers, constituting a quarter of all plastics annually produced globally,^1^ are inherently difficult to recycle. This class of polymers contains covalent cross-links connecting all individual polymer chains together, providing the material class with improved mechanical performance and resistance to solvents and heat.^2^ This comes at the cost of recyclability, as these bonds cannot be disconnected without damaging the polymer permanently. Initiatives to create reversibly bound polymers usually incorporate dynamic, yet directional and localized bonds, creating so-called covalent adaptable networks^3,4^ (CANs) and vitrimers^5,6^ based on dynamic covalent chemistries. Non-covalent interactions are generally weaker than covalent interactions, and often are too sensitive to environmental factors, such as moisture and pH, to strengthen materials in a robust way. For example, ionic interactions, which are strong in vacuum, quickly lose their strength in the presence of water. Polyelectrolyte complexes^7,8^ (PECs), which form by association of oppositely charged polyelectrolytes, are thus very hard and brittle in the absence of water, but very soft and viscous when hydrated. Until recently, there was little evidence that ionic interactions could serve as robust, yet reversible cross-linkers to strengthen plastics.

To address this challenge, we developed ‘compleximers’,^9^ a novel class of hydrophobic PEC materials, in which the ionic interactions are screened by attaching bulky hydrophobic spacers to the charged groups. This introduces hydrophobicity and makes the materials insensitive to water, and at the same time weakens the ionic bonds sufficiently to allow the materials to be reshaped at elevated temperatures. We showed that compleximers can combine the processability and recyclability of thermoplastics with the resistance to solvents of thermosets, demonstrating the potential of ionic interactions as reversible cross-linkers in polymer materials. The ionic groups in compleximers were based on the chemical structure of ionic liquids,^10^ which have bulky ions that lower their glass transition temperature (*T*_g_). Ionic liquids therefore also act as effective plasticizers of compleximers by lowering the material *T*_g_ even further.^11^

While the compleximer concept has proven to be robust, showcasing success across diverse chemical structures, it remains a relatively new area of research and not much is known about their structure–property relationships. To date, much of the experimental and numerical investigations of ionic materials have focused on poly(ionic liquids) (PILs), which are charged polymer melts with counterions,^12–17^ rather than their complexes. Also ionomers, which contain small fractions of charged groups that cluster in ionic aggregates with their counterions^18–20^ have received attention. Polyelectrolyte complexes (PECs) have been studied computationally,^21^ including cases where hydrophobic groups were incorporated,^22^ guided by experimental findings,^23^ but these are focused on the hydrated state. The properties of PECs in the absence of water remain uncharted territory, presenting an opportunity to uncover new insights into their unique behavior and potential applications. Additionally, a simulation approach allows us to understand the effect of changes in the polymer architecture without extensive synthesis approaches.

In this work, we perform coarse-grained molecular dynamics (MD) simulations with a simple bead-spring model to gain deeper insight into the physical and molecular processes underlying the unique properties of compleximers. We investigate complexes of charged polymers with neutral side groups attached to the charged monomers, focusing on the role of the overall charge fraction in the system, through modifying the neutral side chain (SC) length and charge density (CD) on the polymer backbone. We investigate the effect on the structure and dynamics of compleximers, by studying the density, radial distribution function and the number of oppositely charged beads in the first coordination shell, which is a measure for the number of ionic bonds. We then investigate the effect on the relaxation behavior through the intermediate scattering function ISF, from which we extract relaxation times *τ*_*α*_, and construct Angell plots. We also investigate the mean squared displacement MSD, the non-Gaussian parameter *α*_2_, and the Debye–Waller factor 〈*u*^2^〉. We find that diluting the charges in the system, either by decreasing the charge density along the polymer backbones or by increasing the length of the hydrophobic side chains, decreases the glass transition temperature, roughly proportionally to the charge fraction. The glass transition temperature also correlates linearly with the average coordination number of positive–negative charges in the shell of first neighbors. All *τ*_*α*_(*T*_g_/*T*) plots fall on a master curve in Angell plots, showing no significant fragility differences within this simple model. A master curve of rescaled *τ*_*α*_(1/〈*u*^2^〉) values also proves that compleximers obey the fundamental relation between *α*- and *β*-relaxations predicted by the generalized localization model of Simmons *et al.*,^24^ with varying caging exponent showing that highly charged systems are characterized by more anisotropic glassy cages.

## 2 Methods

### 2.1 Modeling compleximers

**Fig. 1:**
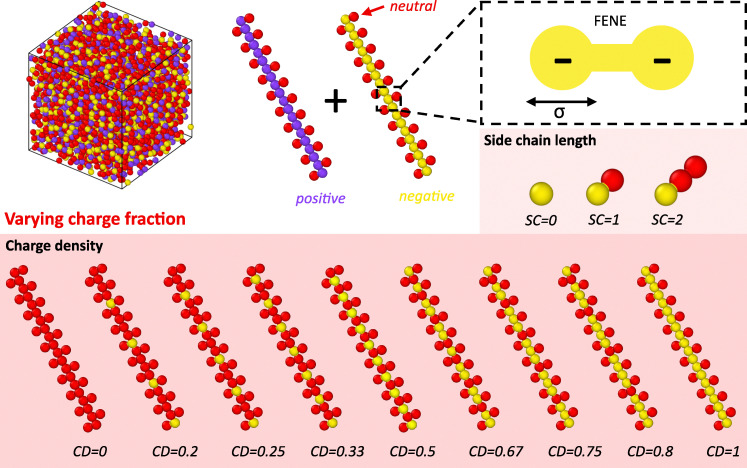
Schematic representation of the coarse-grained polyelectrolytes used for simulating compleximers. The system consists of negatively (yellow) and positively (purple) charged backbones, grafted with neutral (red) beads. All covalent bonds are modeled using the finite extensible non-linear elastic (FENE) potential. We vary the charge fraction of the system by changing the length of the neutral side chain (SC) or the charge density (CD) on the backbone.

To model compleximers, we build 3D simulation boxes of mixtures of oppositely charged polymers. In compleximers, the polymers consist of a neutral backbone with a charged domain attached, which is screened by bulky hydrophobic side chains.^9^ As the charge is relatively close to the backbone (compared to the side chain length), we simplify the chemical structure and create coarse-grained polyelectrolytes with backbones consisting of charged beads (yellow = −1, purple = +1) with neutral beads attached (red = 0) (Fig. 1).

Non-bonded atoms at distance *r* interact through a Lennard-Jones potential with a cutoff of *r*_c_ = 2.5:1
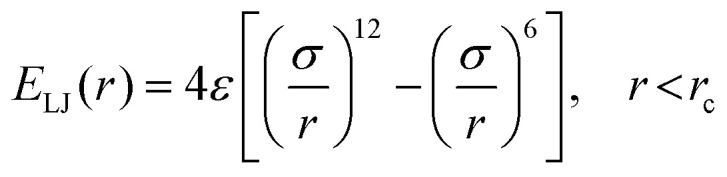
where *ε* is the energy scale of the simulations, which also reflects the strength of the (van der Waals) attraction between the beads, and where *σ* denotes the fundamental length scale of our unit system, corresponding to the bead diameter. All quantities can be represented using the reduced Lennard-Jones units *ε*, *σ*, *m* and the derived time unit 
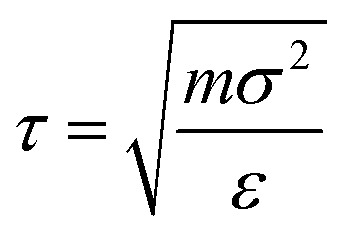
. Each polymer has a backbone length of 20 beads.

All ‘covalently’ linked beads are modeled using the finite extensible non-linear elastic (FENE) potential:^25^2
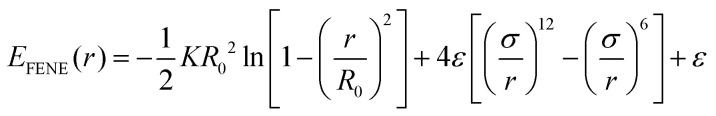


This model combines an attractive first term, with *R*_0_ = 1.5 as the maximum extension of the bond and a bond stiffness *K* = 30, with a repulsive Lennard-Jones (LJ) second term with a cutoff of 
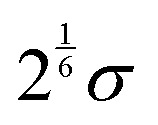
, resulting in an equilibrium bond length of 0.97*σ*.

Electrostatic interactions are captured using the Coulomb potential,3
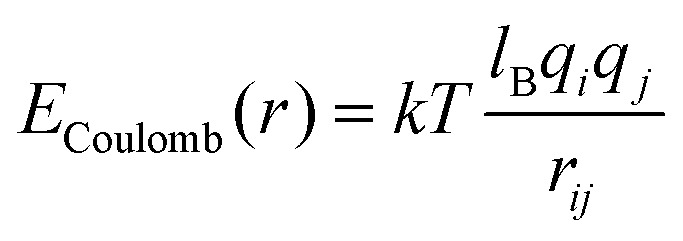
where *q*_*i*_ and *q*_*j*_ are the charge valencies of the respective ions set to ±1. The strength of the Coulomb interaction is set by Bjerrum length *l*_B_ = *e*^2^/*εkT*, where *e* is the elementary charge, *k* = 1 the reduced Boltzmann constant, and *ε* is the dielectric constant. We set *l*_B_ = 10*σ*, producing a relatively strong Coulomb interaction strength in the same range as previous similar studies.^15^ The Coulomb interactions are calculated *via* the particle–particle/particle–mesh (PPPM) method^26^ for all pairs of particles, including bonded beads.

We investigate the effect of neutral attenuator length on the material structure and dynamics by varying the side chain (SC) length from SC = 0 to SC = 2 (Fig. 1), by attaching a single neutral bead or strand of beads per ionic backbone bead. The grafting density is 1, meaning that all charged backbone monomers have a graft when SC > 0. Computational models of these complexes were generated by combining oppositely charged, grafted polyelectrolytes with identical SC lengths. We also model compleximer systems with varying charge density (CD = 0–1) combined with varying side chain length (SC = 0–2). We chose to evenly distribute the charged beads over the polymer chain, starting at the chain end (Fig. 1); these distributions are the same for systems with varying SC length with SC = 0–2. We note that, with these interactions, the liquid-to-solid transition of the system upon variation in temperature is driven by the attractive interactions typical of Lennard-Jones fluids rather than polyelectrolyte complexation, though the presence of charged species strongly influences the local packing and dynamics.

### 2.2 Numerical approach

Using large-scale atomic/molecular massively parallel simulator (LAMMPS),^27^ we perform molecular dynamics simulations on systems of *N*_chains_ = 50 chains of the two respective charges. We use a time step size d*t* = 0.005[*τ*] implementing the NPT ensemble (using Noose–Hoover thermostat and barostat). We begin with an equilibration workflow consisting of a soft potential relaxation, designed to gently resolve overlaps in the initial atomic configuration. This step utilizes a soft interaction potential that ramps up over time, gradually pushing particles apart. Following this, the system transitions to a repulsive Lennard-Jones (LJ) interaction model, which introduces short-range repulsions (cutoff 1.12246) while allowing for controlled isotropic pressure ramping to a final pressure of 1 in reduced units. Finally, the system is subjected to full LJ interactions with a longer cutoff (2.5), gradually ramping down to a pressure of 0.1.

Then follows a relaxation with an emphasis on refining the electrostatic properties through systematic dielectric adjustments. Charges are assigned to specific particle types (1 = neutral, 2 = positive, 3 = negative), establishing the ionic nature of the compleximers. By gradually reducing the dielectric constant (from dielectric constant = 0.8), the system experiences a controlled increase in electrostatic forces. The system is equilibrated at constant pressure (0.1) and temperature (varied between 0.376 and 1.972) in the NPT ensemble during each dielectric step. Following the dielectric relaxation phase, the system undergoes a longer simulation run to fully equilibrate under the final dielectric conditions (final dielectric constant = 0.1) for 1.5 × 10^4^*τ*. For systems with higher relaxation times, this is increased to 1.5 × 10^5^*τ*. In case of a neutral system, the dielectric adjustments are omitted.

Finally we perform a production phase on the relaxed systems to generate trajectory data for 3 × 10^4^*τ* for fast relaxing systems and 1 × 10^5^*τ* for slowly relaxing systems. The trajectories are saved following a log-linear time series, and at each timestep, 100 samples are drawn from the trajectory and averaged to compute the relevant quantities.

## 3 Results

### 3.1 Structural properties

We introduced the concept of compleximers experimentally by synthesizing three sets of materials with progressively moderated ionic groups.^9^ We showed that an adequate level of moderation resulted in thermally processable materials. However, these experiments did not reveal how the attachment of bulky side chains affects the interactions between charged moieties, and how the changes in processability are related to changes in the nanostructure of the materials. Experimentally, the moderation of the charge interactions was achieved by separating the charges through the attachment of side chains; all experimental compleximers have a charge density of 1, meaning they contain a charged moiety on every monomer. However, it is interesting to study whether reducing the charge density along the polymer backbone has a similar effect as adding non-charged side chains. We therefore investigate the effect of lowering the charge fraction (defined as = *N*_beads,charged_/*N*_beads,total_) in the system using both strategies.

#### 3.1.1 Ionic interactions and material cohesion

**Fig. 2:**
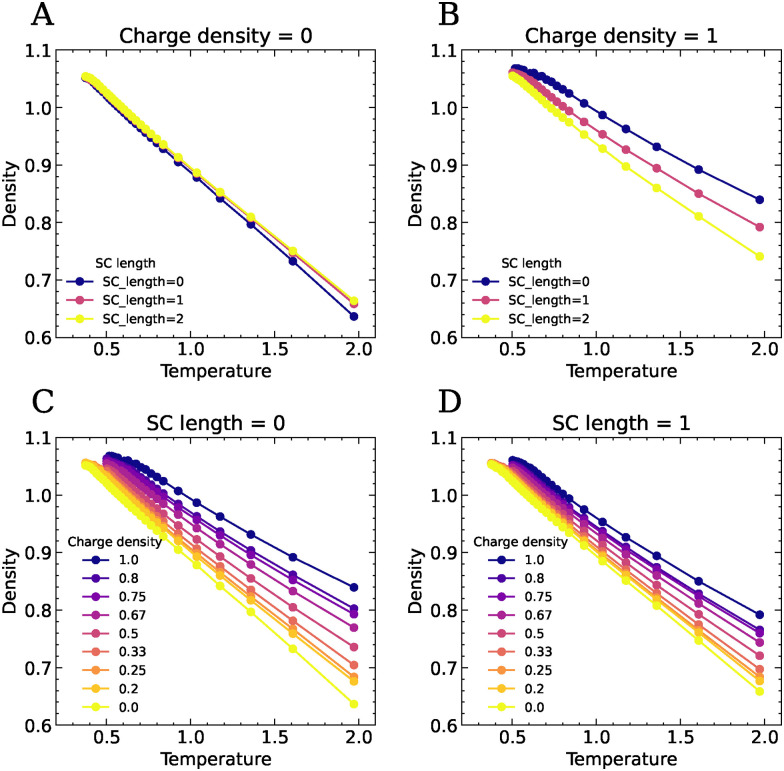
The density as a function of temperature for systems with varying side chain length SC for (A) neutral polymers and (B) fully charged polymers, and for systems with varying backbone charge density for (C) SC = 0 and (D) SC = 1.

As shown in Fig. 2A and B, charged systems exhibit higher densities (number of beads per unit volume) and lower thermal expansion coefficients (represented by the slope of the plots) compared to their neutral counterparts (CD = 0). This effect is not pronounced at low temperature due to the packing limits of steric repulsion, but becomes prominent at higher temperature. With increasing side chain length, the density of the charged system decreases while the expansion coefficient rises, reflecting the reduction in Coulomb forces resisting expansion. By contrast, neutral systems with varying side chain lengths exhibit nearly constant densities and expansion coefficients. A similar trend is obtained by changing the backbone charge density: as the charge denisty is reduced, the density decreases and the thermal expansion coefficient increases (Fig. 2C and D). This trend indicates that the ionic interactions keep the material together and increase the cohesive energy of the system, which has also been observed in computer simulations of polyionic liquids with increasing strength of the ionic interactions through varying the Bjerrum length.^15,20^ The effect of changing the backbone charge density is less pronounced for compleximers with longer neutral spacers (Fig. S1).

#### 3.1.2 Separation and coordination of opposite charges

The observed density decrease in charged systems resulting from SC incorporation can be explained by a decrease of the cohesive energy. This could be due to an increased separation distance between opposite charges as the side chains hinder approach of the charged beads. To investigate the separation distances between beads, we calculate the partial radial distribution functions *g*_ab_(*r*), which give the average number of neighbors of type b in a shell at distance *r* around particles of type a, capturing the degree of order and correlation in the system:4
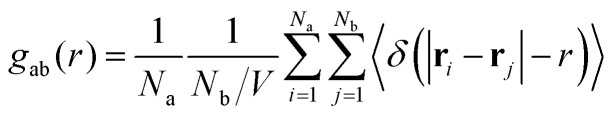


**Fig. 3:**
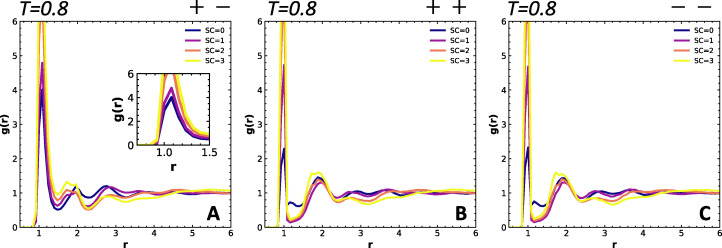
The partial radial distribution function (*g*_ab_(*r*)) for compleximers with varying SC length for the pair interactions at *T* = 0.8 for (A) +− pairs, where the inset shows that the width of the first peak increases with increasing side chain length, suggesting that the overall separation distance between the opposite charges increases; (B) ++ pairs; and (C) −− pairs.

This equation is normalized so that the *g*(*r*) becomes 1 at large separation distances. We quantify the radial distribution for all pairs of particle types. Results for the +−, ++, and −− pairs at *T* = 0.8 are shown in Fig. 3, while those involving neutral beads (00, 0+, and 0−) are shown in Fig. S2. We find that the first peak in the *g*(*r*) of the +−, ++ and −− interaction increases in height as the SC length increases, which is likely due to the normalization; the number of charged groups at large distances scales with the fraction of charged groups and thus decreases with SC length. By normalizing such that *g*(*r*) approaches 1 at large *r*, the graphs for higher SC lengths are shifted relatively upwards. A shift of the first peak of *g*_+−_(*r*) to higher *r* would indicate an increase in the charge separation distance, but it is difficult to determine whether the broadening of the peak (Fig. 3A, inset) results from the increase in peak height or change in separation distance. We show in Fig. S3 a similar trend as a result of the lowering of the backbone charge density. The primary peak broadening and the increase in peak height is more pronounced in the system without neutral side chains. We also find that the second and third particle shells shift to larger distances and become smaller, indicating that the distance between the opposite charges, also those further away, increases. Li *et al.* report similar findings in ionomers with varying interaction strength,^20^ where the primary peak shifts to higher *r* when the Bjerrum length *l*_B_ decreases (weaker interaction). The pairwise interactions between like charges are not heavily influenced by the presence of the side chain, and are dominated by the fact that they are connected covalently (Fig. 3B and C). There are also no noteworthy effects on the pairwise interactions involving neutral beads (Fig. S2A). We can therefore conclude that changes in the radial distribution functions, especially involving charged beads, are very subtle across all cases, and challenging to interpret.

**Fig. 4:**
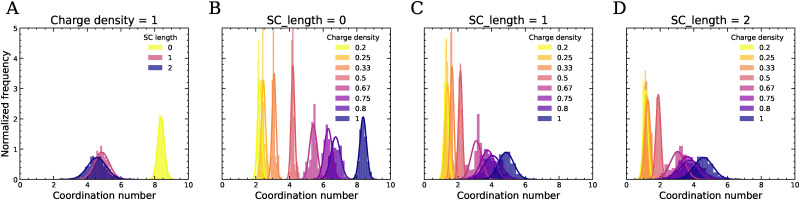
The +− coordination number in the first coordination shell at *T* = 0.8 for complexes with (A), CD = 1 and varying side chain length. (B), SC = 0, (C), SC = 1 and (D), SC = 2 with varying charge density. The number of +− contacts drops significantly upon increasing the length of the neutral SC. The number of oppositely charged neighbors in the first coordination shell decreases with decreasing charge density. Peak positions and variance are reported in Tables S1 and S2.

Since variations in *g*_ab_(*r*) are rather subtle, we analyze them further by calculating the coordination numbers *C*_ab_ in the first coordination shell of each charged particle by integrating the radial distribution function *g*_ab_(*r*) over the distance range corresponding to the first coordination shell:5
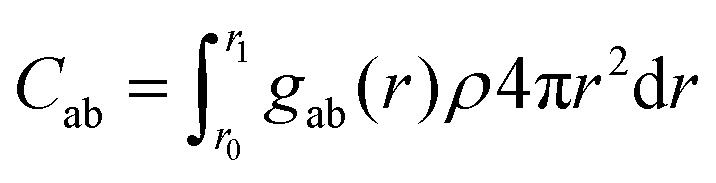
where *g*_ab_(*r*) is the radial distribution function, *ρ* is the number density of atoms, and *r*_0_ and *r*_1_ define the limits of the first coordination shell, where *r*_0_ = 0.1 and *r*_1_ is the first minimum in *g*(*r*). The factor 4π*r*^2^ represents the spherical volume element. In particular, the coordination number *C*_+−_, giving the number of oppositely charged beads in the first coordination shell, is a measure for the number of ionic bonds in the material. In Fig. 4 we show histograms of *C*_+−_ at *T* = 0.8 for systems with different charge density and side chain length. Attaching neutral beads to the charged backbones results in a dramatic decrease in *C*_+−_ (Fig. 4A), indicating that the neutral beads disrupt the local ordering around the charged beads and reduce the number of ion pairs. As the neutral side chains grow longer, the decrease in *C*_+−_ becomes more subtle, indicating that longer side chains further diffuse the distribution of charged beads, but with diminishing effect. This suggests a limit in the disruption of local structure beyond which additional SC length has little impact. This is expected, as the first neutral spacer directly occupies the first shell of neighbours from which the coordination number is calculated. With decreasing charge density, a significant decrease in the number of neighboring opposite charges occurs at SC = 0 (Fig. 4B). Separating the charges along the backbone thus affects the spatial distribution of opposite charges in the first coordination shell. When side chains are attached (Fig. 4C and D), *C*_+−_ at CD = 1 is already much lower, but further reductions in charge density still result in a clear decrease in the number of +− contacts. The +− coordination numbers of systems with SC = 1 and SC = 2 are similar, highlighting the diminishing impact of further increasing the SC length. The attachment of neutral spacers, whether as a side chain or in the backbone, thus has a subtle effect on the separation distance between the nearest oppositely charged beads, but primarily reduces the number of neighboring oppositely charged beads.

### 3.2 Dynamics

#### 3.2.1 Relaxation and fragility

Changes in the material structure are closely related to the dynamic properties, which constitute the defining feature of glass-forming systems. From the production trajectories, we calculate the self-part of the intermediate scattering function (ISF) averaged over all particles,6
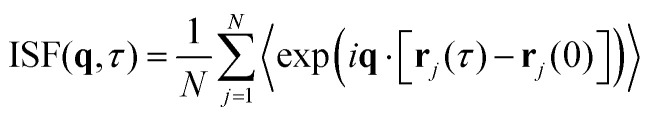
where the wave vector, with magnitude *q* = 2π/*λ*, is a measure for the probed length scale. Here we choose *q* = 7.1, which is roughly the peak of the static structure factor corresponding to the length scale of the first-neighbor's cage. The ISF, shown in Fig. S4–S6 for the different systems at different temperatures, describes the loss of correlation of the particle positions as a function of time, due to diffusion or other dynamic processes. For glassy systems, especially at low temperatures, the intermediate scattering function shows two distinct relaxation processes. The fast (*β*) relaxation arises from local ‘cage’ dynamics, such as vibrations or rattling motions of particles within their immediate surroundings.^28,29^ The slow relaxation at higher *τ*, also called the *α* relaxation, represents cage-breaking events that drive structural relaxation, and is often associated with cooperative processes involving many particles. At low temperature, these two processes are well-separated in time due to the constrained motion and the presence of energy barriers that limit large-scale dynamics, but they are known to be connected.^24,28,30^ At high temperatures, the ISF transitions to displaying a single relaxation time since the thermal energy becomes sufficient to overcome local barriers, accelerating both local and collective motions. As a result, the distinction between *β* and *α* relaxation processes blurs and local and collective dynamics converge into a single, diffusive relaxation process. All the ISF curves are reported for completeness in (Fig. S4–S6), but due to the extensive number of simulations performed, we report here only the key dynamical quantities extracted from them.

**Fig. 5:**
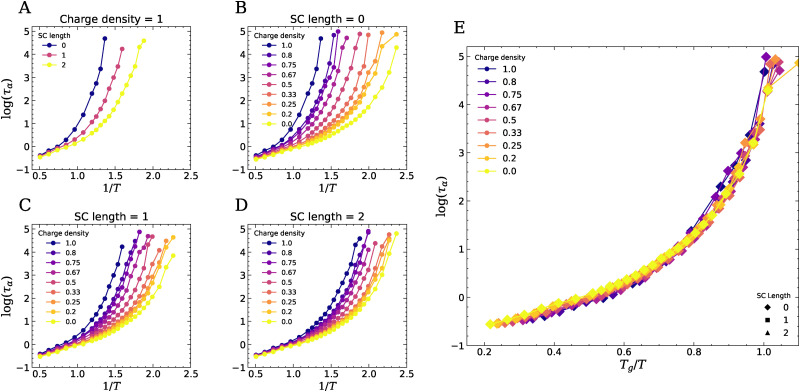
The *α*-relaxation time of compleximers as a function of temperature. Data reported for fixed charge density (CD = 1) and varying SC length (A), or varying charge density and fixed side chain length of 0 (B), 1 (C), or 2 (D). (E) The data can be rescaled on a master curve by rescaling the horizontal axis with the *T*_g_ of each system, giving a universal fragility plot.

**Fig. 6:**
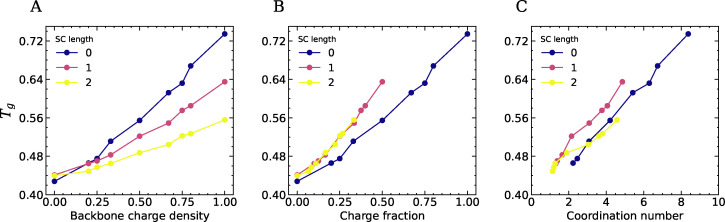
The glass transition temperature plotted as a function of (A) the charge density of the polymer backbones for three different SC length, (B) the charge fraction in the system (the fraction of beads in the system that carries a charge), and (C) the +− coordination number.

We calculate the caging relaxation time *τ*_*α*_ as the time over which the ISF decays to 0.2,^15^ and plot *τ*_*α*_ as a function of 1/*T* for systems with varying molecular parameters, see Fig. 5A–D. These curves show the characteristic slowing down of the dynamics with decreasing temperature. All curves deviate from Arrhenius behaviour and show a steeper increase of the relaxation time as the temperature is lowered towards the glass transition. This is well-known for polymeric glass formers, and is often described using semi-empirical relations such as the Williams–Landel–Ferry (WLF) equation^31^ or the Vogel–Fulcher–Tamman (VFT) equation.^32–34^ We find that both the incorporation of neutral side chains (Fig. 5A) and decreasing the charge density of the backbone (Fig. 5B–D) accelerate the dynamics of the compleximers, highlighting how ionic interactions restrict the mobility of polymer segments. We define the glass transition temperature *T*_g_ as the temperature at which *τ*_*α*_ = 10^4^[*τ*], beyond which the system falls out of equilibrium within the simulation timescale. Clearly, the *T*_g_ shifts to lower values as the charge density of the polymers decreases or as the length of the side chains increases (Fig. 6A). Systems without neutral side chains have the highest *T*_g_, and *T*_g_ decreases with increasing SC length, which is in line with our experimental findings.^9^ Lowering the charge density to 0 eventually leads to approximately the same dynamics irrespective of the SC length. We note that the changes in dynamics and *T*_g_ with changing charge fraction appear to follow changes in the density and the expansion coefficient (Fig. 2A). For example, at SC = 0, both the changes in dynamics and those in density and the expansion coefficient with varying charge density are much more pronounced than at SC = 2. This suggests that the temperature-dependent changes in dynamics are correlated with changes in density.

To further investigate the effect of electrostatic interactions on *T*_g_, we plot the *T*_g_ as a function of the total charge fraction (= *N*_beads,charged_/*N*_beads,total_) in Fig. 6B. We find that *T*_g_ increases more or less linearly with increasing charge fraction. However, systems with neutral side chains exhibit higher *T*_g_ values at the same charge fraction than systems without neutral side chains, suggesting that spacing charges along the backbone of linear polyelectrolytes is more effective at lowering *T*_g_ than adding hydrophobic side chains. We also plot *T*_g_ as a function of the +− coordination number, defined as the number of oppositely charged beads in the first coordination shell, revealing a clear correlation between *T*_g_ and the number of ion pairs (Fig. 6C). Our findings of a lowering of *T*_g_ upon weakening the ionic interactions is in line with simulation results on polyionic liquids from Bocharova *et al.*,^14^ who also found that *T*_g_ decreases as the number of charges per unit volume decreases. We believe these general findings will persist also at different molecular weight of the polymer chains, but it would be interesting in future work to see how the trends are affected by the packing frustration induced by entanglements.

Using the values for *T*_g_ obtained from the simulations, we rescale the horizontal axes to construct Angell plots (Fig. 5E).^35,36^ We find that all curves collapse on one master curve, which indicates that the fragility is not affected by the side chain length nor by the charge density. This is not in agreement with our previous experimental findings that showed a lower fragility for charged polymers,^37^ and we attribute the discrepancy to the oversimplifications of the bead-spring model. Future work including chemistry-specific features such as different bead sizes and masses, cross-interactions, and varying rigidity of different chain segments, which are known to affect fragility according to the generelazied entropy theory,^38,39^ will be crucial to further capture the glass-forming features of this new class of materials.

#### 3.2.2 Mean squared displacement (MSD) and particle localization

The mean squared displacement (MSD) reflects how far particles move on average over time. It is defined as:

7 MSD(*t*) = 〈|**r**(*t*) − **r**(0)|^2^〉

with *r*(*t*) the position at time *t* and *r*(0) the position at time *t* = 0 and where the average is taken over all particles and all initial times. As for the ISFs, we report the full MSD curves of our systems in the SI, Fig. S7–S9. They all have three distinct regimes:^40^ at short timescales the particles move ballistically without encountering other particles, leading to MSD ∼ *t*^2^; this is followed by a sub-diffusive regime with MSD ∼ *t*^*α*^, *α* < 1, where the particles can only move locally due to the ‘caging’ effect; and finally, at long times the particles can escape from their cage, leading to a diffusive regime where MSD ∼ *t*. As the temperature decreases, the subdiffusive regime becomes more pronounced, indicating stronger localization of the particles in their cages. This is accompanied by strong dynamic heterogeneities (Fig. S10), a hallmark feature of glassy dynamics.^41,42^

**Fig. 7:**
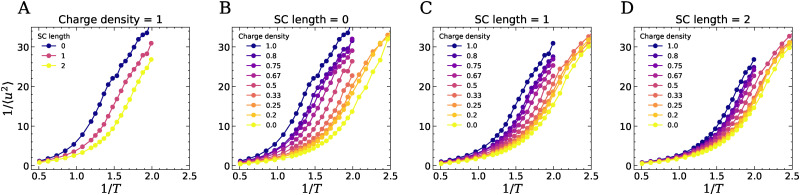
The inverse Debye–Waller factor (1/〈*u*^2^〉) of compleximers as a function of temperature. Data reported for fixed charge density (CD = 1) and varying SC length (A), or varying charge density and fixed side chain length of 0 (B), 1 (C) and 2 (D).

In Fig. 7 we report the inverse of the Debye–Waller factor 〈*u*^2^〉 as a function of 1/*T*, extracted from the MSD curves as the value of the MSD at the caging time t* ∼ 3 where the slope of the MSD has its lowest value. The Debye–Waller factor is a measure for the size of the confining cage of the particles. It has been extensively reported in the simulation literature as a key feature of the glassy caging dynamics, inversely correlating with *τ*_*α*_ for a wide class of systems^43,44^ and also with the elastic modulus of thermoplastics and polymer composites below *T*_g_.^45,46^ From the figure, we can see that 1/〈*u*^2^〉 shows similar behavior as *τ*_*α*_: as the temperature decreases, the particles become more confined so that 1/〈*u*^2^〉 increases, and the slope at which this happens increases with decreasing temperature. The increase of 1/〈*u*^2^〉 shifts to lower temperatures as the charge density decreases, or as the length of the neutral side groups increases, which again implies that the weakening of the ionic interactions enhances particle dynamics and lowers the *T*_g_.

Leporini *et al.* established a universal correlation between the structural relaxation time and the Debye–Waller factor in glass-forming liquids,^30,47^ later refined by Simmons *et al.* in their generalized localization model based on free volume arguments.^24^ In the most recent formulation,^43^ this scaling takes the form

**Fig. 8:**
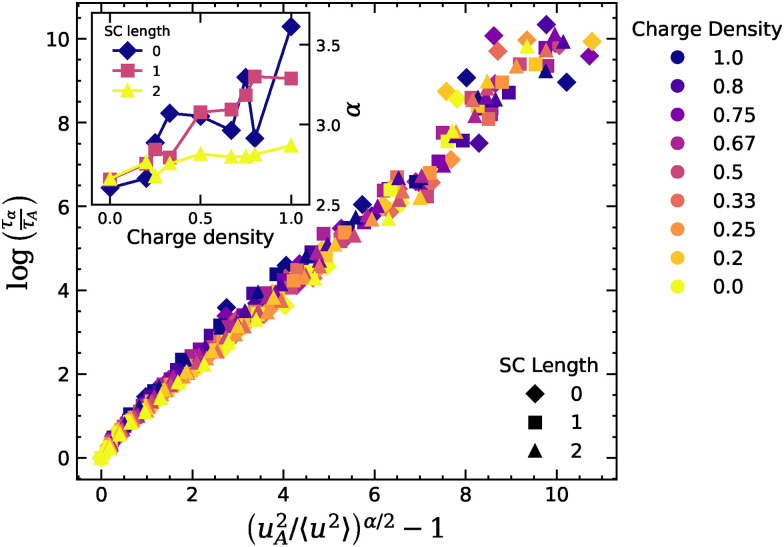
Collapse of the structural relaxation time *versus* the Debye Waller factor according to eqn (8) for all systems with varying CD and SC length. The inset shows the value of the exponent *α* as a function of the backbone charge density for different SC length.

8 *τ*_*α*_ = *τ*_A_ exp[(〈*u*_A_^2^〉/〈*u*^2^〉)^*α*/2^ − 1],

where *τ*_A_ and 〈*u*_A_^2^〉 are the values of the relaxation time and Debye–Waller factor measured at the Arrhenius temperature *T*_A_ signaling the onset of glassy dynamics. For the system with SC = 0 and CD = 0, *T*_A_ is taken as the highest temperature at which a linear fit of log(*τ*_*α*_) *vs.* 1/*T* yields a coefficient of fit *R*^2^ > 0.99. For the other systems, *T*_A_ is taken as the temperature at which *τ*_*α*_ is closest to that of SC = 0, CD = 0. The only remaining free parameter is then the exponent *α*, roughly expected to be around 3 for spherical cages and to increase in the presence of cage anisotropy, based on free volume arguments.^24^Fig. 8 shows that all systems fall on a master curve once rescaled appropriately with a system-dependent *α* exponent, reported in the inset of the figure. From the fit, we obtain a value of *α* ∼ 2.6 for neutral systems, which then increases up to ∼3.6 for the CD = 1, SC = 0 systems with the highest charge fraction. The increase of *α* with charge density is more modest for SC = 1, 2. The collapse of the data on this master curve shows that compleximers simulated with simple bead-spring models also obey the universal scaling between caging length scales and slow relaxation dynamics, and that the presence of ionic bonds makes the cages less isotropic (as signified by the higher *α* value^24^). Note that this shift is also apparent in the unscaled data, see Fig. S11, and does not depend on the fitting procedure used to find the values of *α*. This could be an important hint to understand our experimental data showing that compleximers have strong glass-forming features, but we expect that higher resolution models will be needed to directly capture this fragility effect.

## Conclusions

When designing compleximers, we have previously shown experimentally that an increase in the length of the neutral domain leads to a lowering of the *T*_g_, which enables processing.^9^ Our simulations reported in this work support this experimental finding and show that the lowering of *T*_g_ is related to a reduction in the number of direct contacts between oppositely charged groups, leading to an overall decrease of the cohesive energy. Additionally, our simulations show that a lower *T*_g_ can also be achieved by reducing the charge density on the polymer backbone, even in the absence of a neutral side chain. Since the neutral attenuator plays a crucial role in enhancing the hydrophobicity of compleximers, a combination of a lower charge density and presence of hydrophobic domains may offer the best balance for making compleximers with desired properties. Furthermore, increasing the length of the neutral spacer beyond a certain point may not be particularly effective in combination with reduced charge densities, as our simulations indicate that the effect on the +− coordination number does not decrease substantially with further spacer elongation. To this end, increasing the number of side chains per bead may be more effective. Our simulations thus provide guidelines to further tune the *T*_g_ of compleximers experimentally.

In this work we have only considered symmetric systems, in which both polymers have identical charge density and side chain length. Experimentally, also asymmetric mixtures were investigated, in which only one of the polymers carried bulky side groups. These so-called ‘half-screened’ compleximers also showed a lowering of *T*_g_, but much less pronounced than the symmetric mixture.^9^ Moreover, we have shown experimentally that the compleximers can be plasticized further by incorporating ionic liquids.^11^ Future simulation work may provide more detailed insights in these observations. Another avenue for future work is to explore the interaction of compleximers with solvents. The hydrophobic groups on the charged polymers make compleximers insensitive to water and other solvents, and simulations may help to unravel how this solvent-resistance depends on the balance between charged and hydrophobic groups.

Despite the simplicity of the current simulations, we show that model compleximers obey the universal scaling between *τ*_*α*_ relaxation time and *β*-relaxation rattling motion 〈*u*^2^〉 expected from the generalized localization model,^24,43^ with free volume exponents indicating increasing anisotropy of the glassy cages with increasing charge fraction in the system. In future simulations, further modifications to the compleximer architecture are possible, including variations in chain bending energy,^13,14^ grafting density, and the placement of grafts. Additionally, the positioning of ionic groups, such as relocating them to the side chains, could be explored to more closely resemble the chemical structure of compleximers. Other architectural variations may include the charge sequence, which could be random^20^ or uniformly spaced (for instance in blocks). Furthermore, the ionic interaction strength can be adjusted by modifying the Bjerrum length^15^ or the dielectric constant.^14^

To better capture the chemical heterogeneity of the experimental system, it may be necessary to explore chemistry-specific simulations to further illuminate the underlying differences in fragility. The bead-spring model systems presented here provide a solid foundation, but they also underscore the complexity of the underlying mechanisms governing fragility, which cannot be fully captured by this relatively simple model alone, as all Angell plots collapse on the same curve. This highlights the exciting potential to explore the intricate, still-unexplored fundamental processes underlying this unique behavior.

## Author contributions

SvL, AG, and JvdG conceived the project; NV and AG performed the programming and code testing; SvL and NV performed the simulations. SvL, NV, and AG performed data analysis. SvL and JvdG wrote the original draft, which was reviewed and edited by all authors.

## Conflicts of interest

There are no conflicts to declare.

## Supplementary Material

SM-022-D5SM01081K-s001

## Data Availability

Supplementary information is available. See DOI: https://doi.org/10.1039/d5sm01081k. The LAMMPS code used to perform the simulations is available at: https://github.com/giuntoli-group/compleximers-structure-dynamics.
