# Supplementary material for: A molecular dynamics study of the structure and dynamics of screened polyelectrolyte complex materials
Source: Soft Matter. 2026 Jan 14;22(5):1195–204. doi: 10.1039/d5sm01081k (PMC12814932; doi:10.1039/d5sm01081k)
Supplement: SM-022-D5SM01081K-s001 [file SM-022-D5SM01081K-s001.pdf]

Supplementary Information (SI) for Soft Matter.
This journal is © The Royal Society of Chemistry 2026

# Supplementary Information for: A molecular dynamics study of the structure and dynamics of screened polyelectrolyte complex materials

Sophie G.M. van Lange[1], Nayan Vengallur[2], Andrea Giuntoli*[2], and Jasper van der Gucht*[1]

[1]Physical Chemistry and Soft Matter, Wageningen University, 6708 WE, Wageningen, The Netherlands. Email: jasper.vandergucht@wur.nl

[2]Zernike Institute for Advanced Materials, University of Groningen, 9747 AG, Groningen, The Netherlands. Email: a.giuntoli@rug.nl

## Supplementary Information Contents

**Supplementary Figures** **2**

S1. Density versus temperature for SC=2 . . . . . . 2

S2-3. Radial distribution functions . . . . . . 3

S4-6 Intermediate scattering functions. . . . . . 4

S7-9. Mean-squared displacements . . . . . . 7

S10. Non-Gaussian parameter . . . . . . 10

S11. Relaxation time versus Debye-Waller factor . . . . . . 11

## Supplementary Figures

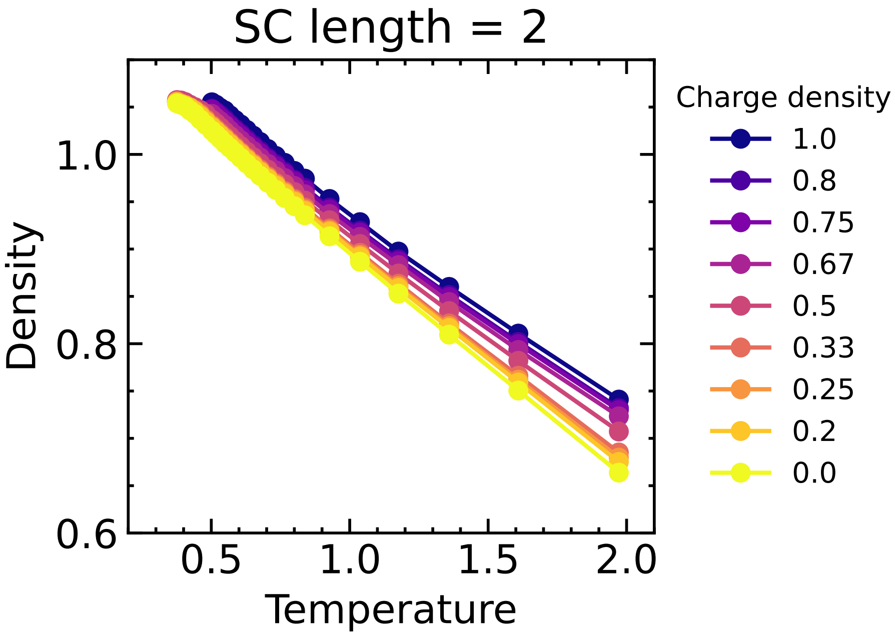


**Fig. S1**: The density as a function of temperature for compleximers with $SC = 2$ and varying charge density.

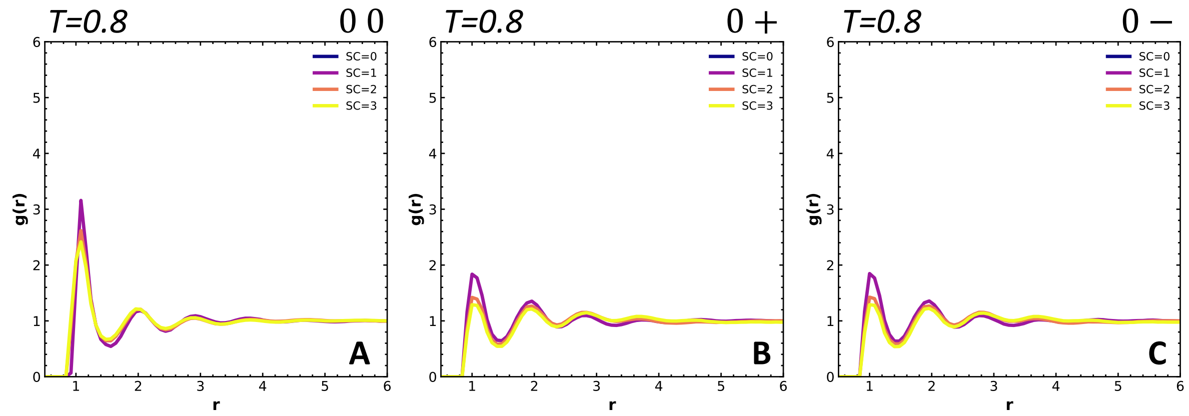


**Fig. S2**: The radial distribution function ($g_{ab}(r)$) of compleximers ($CD = 1$) with varying $SC$ length at $T = 0.8$ for the pair interactions **(A)**, 00. **(B)**, 0+. **(C)**, 0−.

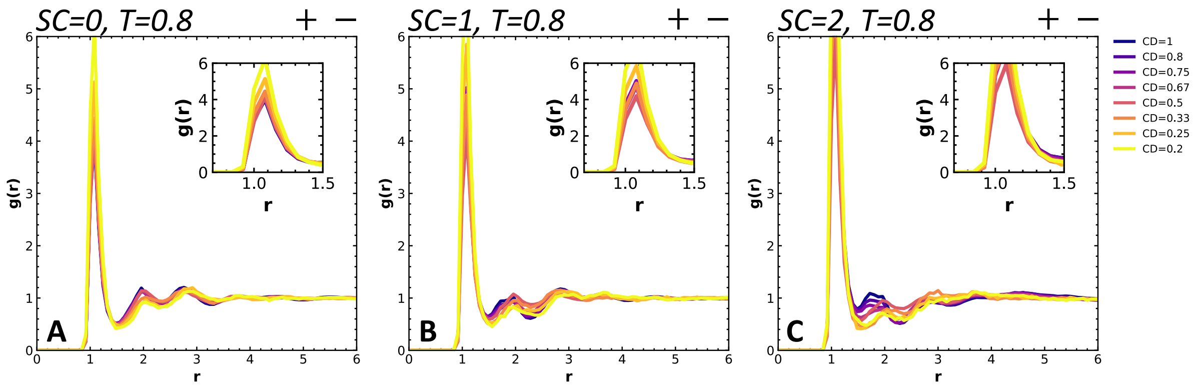


**Fig. S3**: The radial distribution function ($g_{+-}(r)$) of compleximers with varying $CD$ and $SC$ length for the $+-$ pair interactions at $T = 0.8$, for **(A)** $SC = 0$, **(B)** $SC = 1$, and **(C)** $SC = 2$. The inset shows the broadening of the first peak.

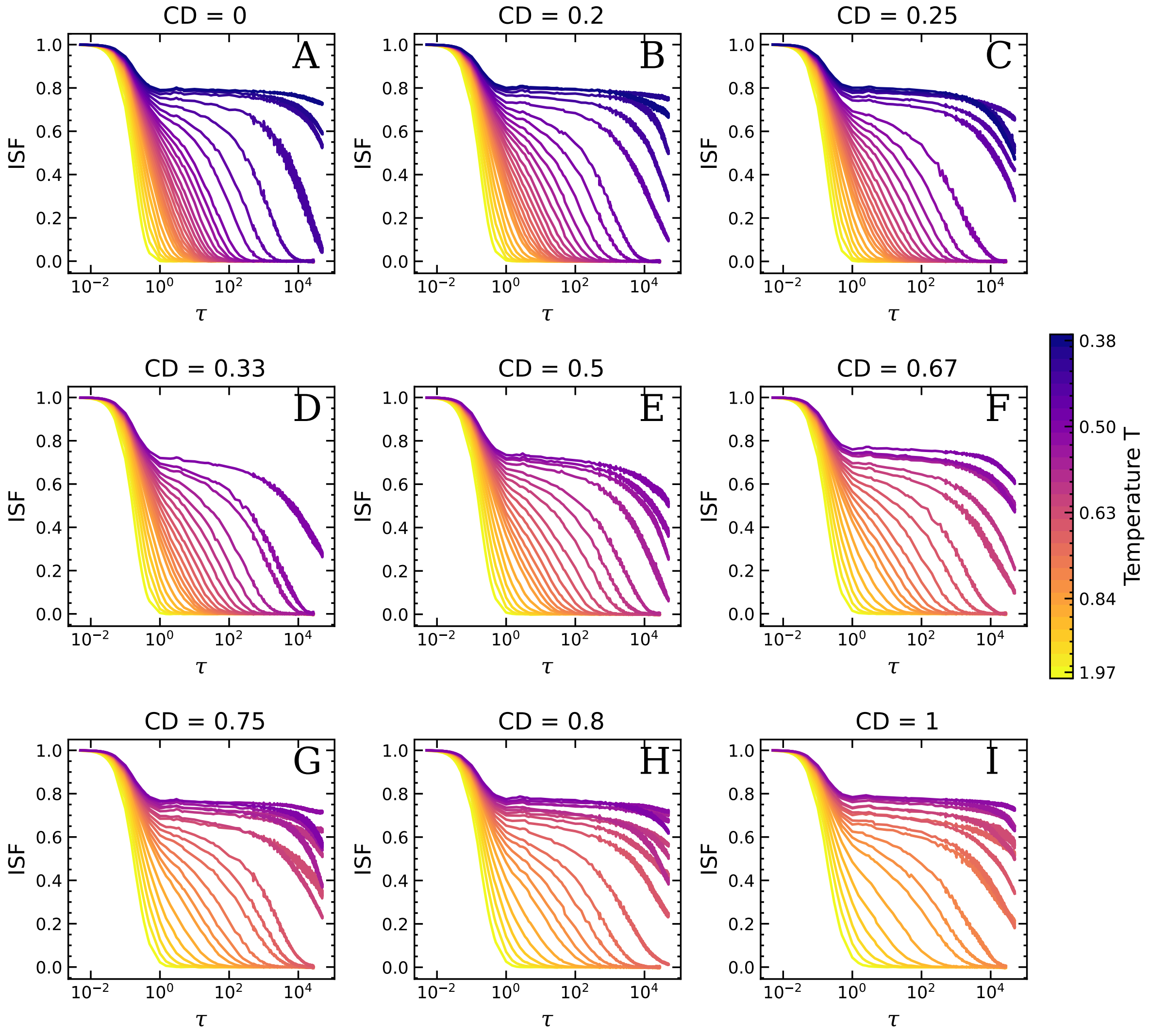


**Fig. S4**: The intermediate scattering function of compleximers with varying charge density $CD$, for $SC = 0$. **(A)**, $CD = 0$. **(B)**, $CD = 0.2$. **(C)**, $CD = 0.25$. **(D)**, $CD = 0.33$. **(E)**, $CD = 0.5$. **(F)**, $CD = 0.67$. **(G)**, $CD = 0.75$. **(H)**, $CD = 0.8$. **(I)**, $CD = 1$. The ISF of some temperatures below $T_g$ do not decay to 0.2, these curves are omitted from the $\tau_\alpha$ calculations.

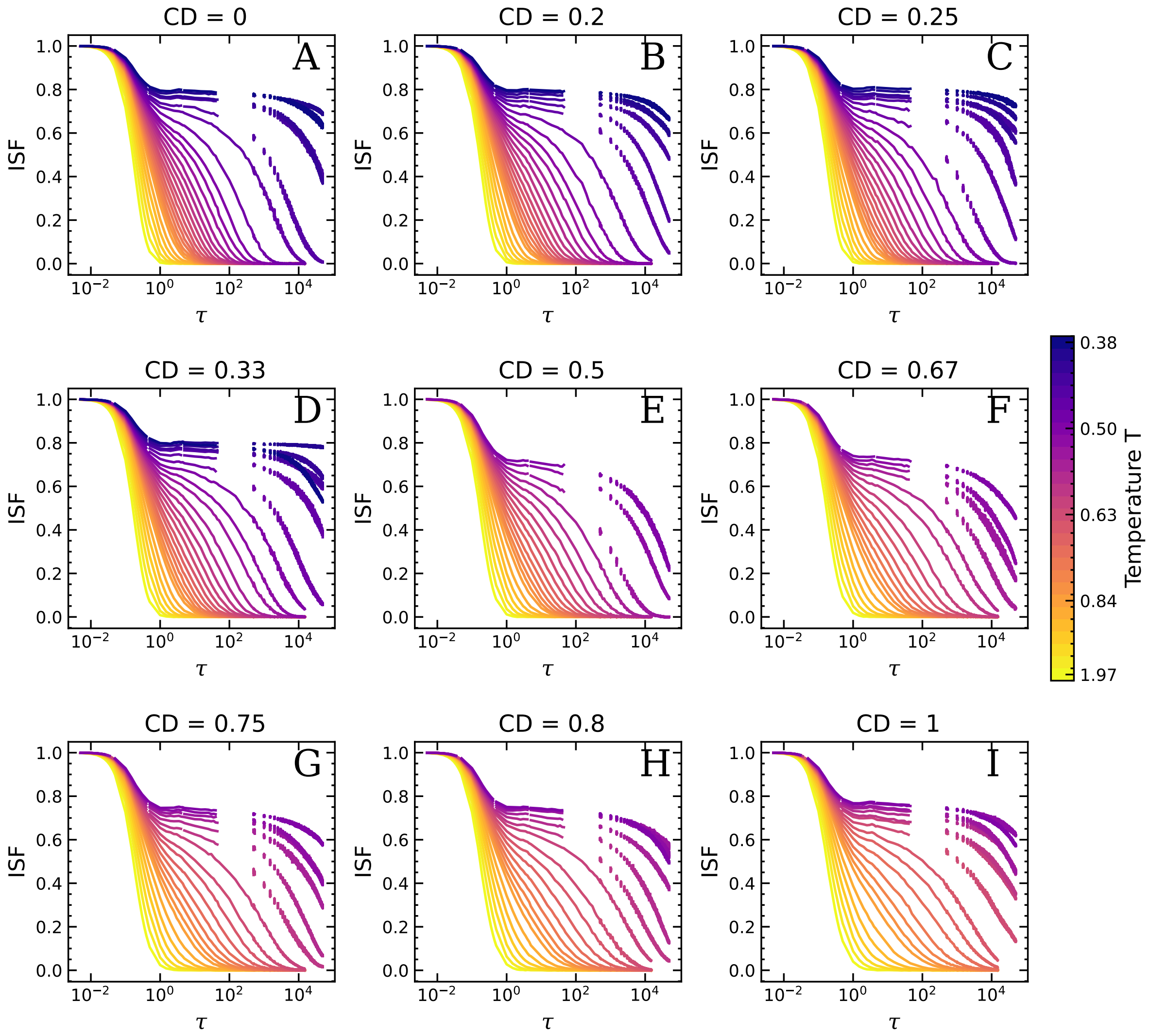


**Fig. S5**: The intermediate scattering function of compleximers with varying charge density $CD$, for $SC = 1$. **(A)**, $CD = 0$. **(B)**, $CD = 0.2$. **(C)**, $CD = 0.25$. **(D)**, $CD = 0.33$. **(E)**, $CD = 0.5$. **(F)**, $CD = 0.67$. **(G)**, $CD = 0.75$. **(H)**, $CD = 0.8$. **(I)**, $CD = 1$.

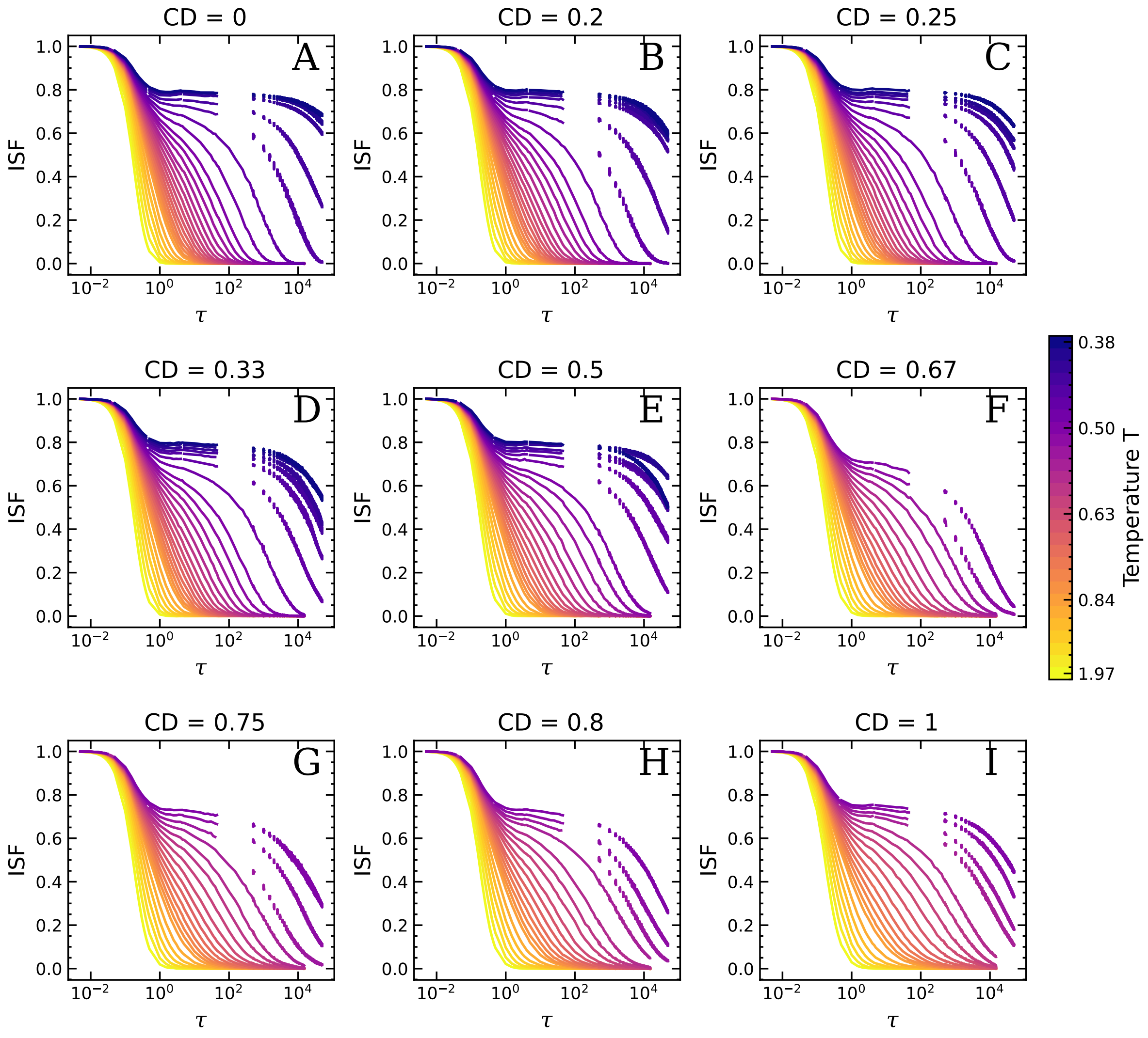


**Fig. S6**: The intermediate scattering function of compleximers with varying charge density $CD$, for $SC = 2$. **(A)**, $CD = 0$. **(B)**, $CD = 0.2$. **(C)**, $CD = 0.25$. **(D)**, $CD = 0.33$. **(E)**, $CD = 0.5$. **(F)**, $CD = 0.67$. **(G)**, $CD = 0.75$. **(H)**, $CD = 0.8$. **(I)**, $CD = 1$. The ISF of some temperatures below $T_g$ do not decay to 0.2, these curves are omitted from the $\tau_\alpha$ calculations.

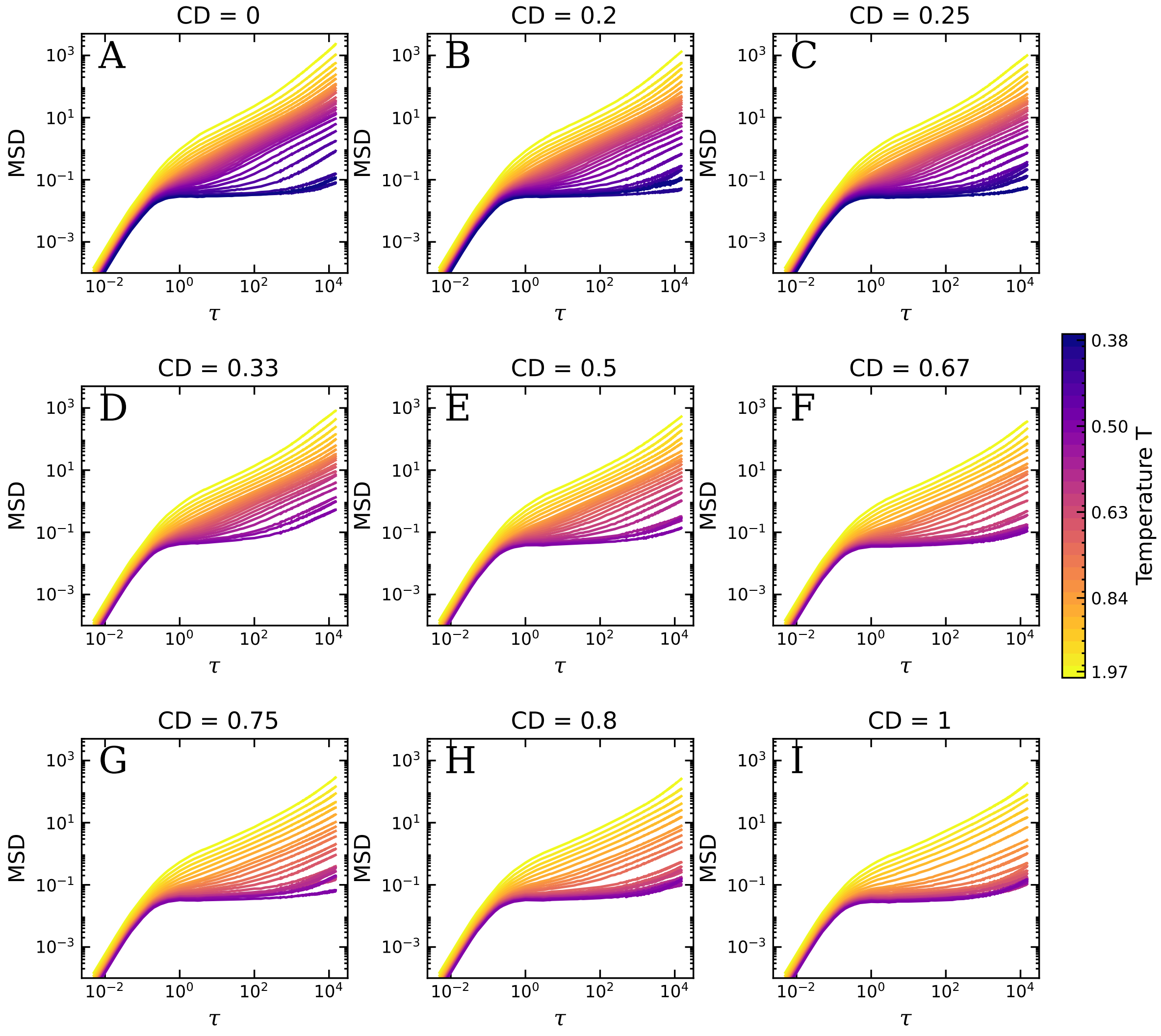


**Fig. S7**: The mean squared displacement ($MSD$) of compleximers with varying charge density ($CD$) at a side chain length $SC = 0$. **(A)**, $CD = 0$. **(B)**, $CD = 0.2$. **(C)**, $CD = 0.25$. **(D)**, $CD = 0.33$. **(E)**, $CD = 0.5$. **(F)**, $CD = 0.67$. **(G)**, $CD = 0.75$. **(H)**, $CD = 0.8$. **(I)**, $CD = 1$. The ISF of some temperatures below $T_g$ do not decay to 0.2, these curves are omitted from the $\tau_\alpha$ calculations.

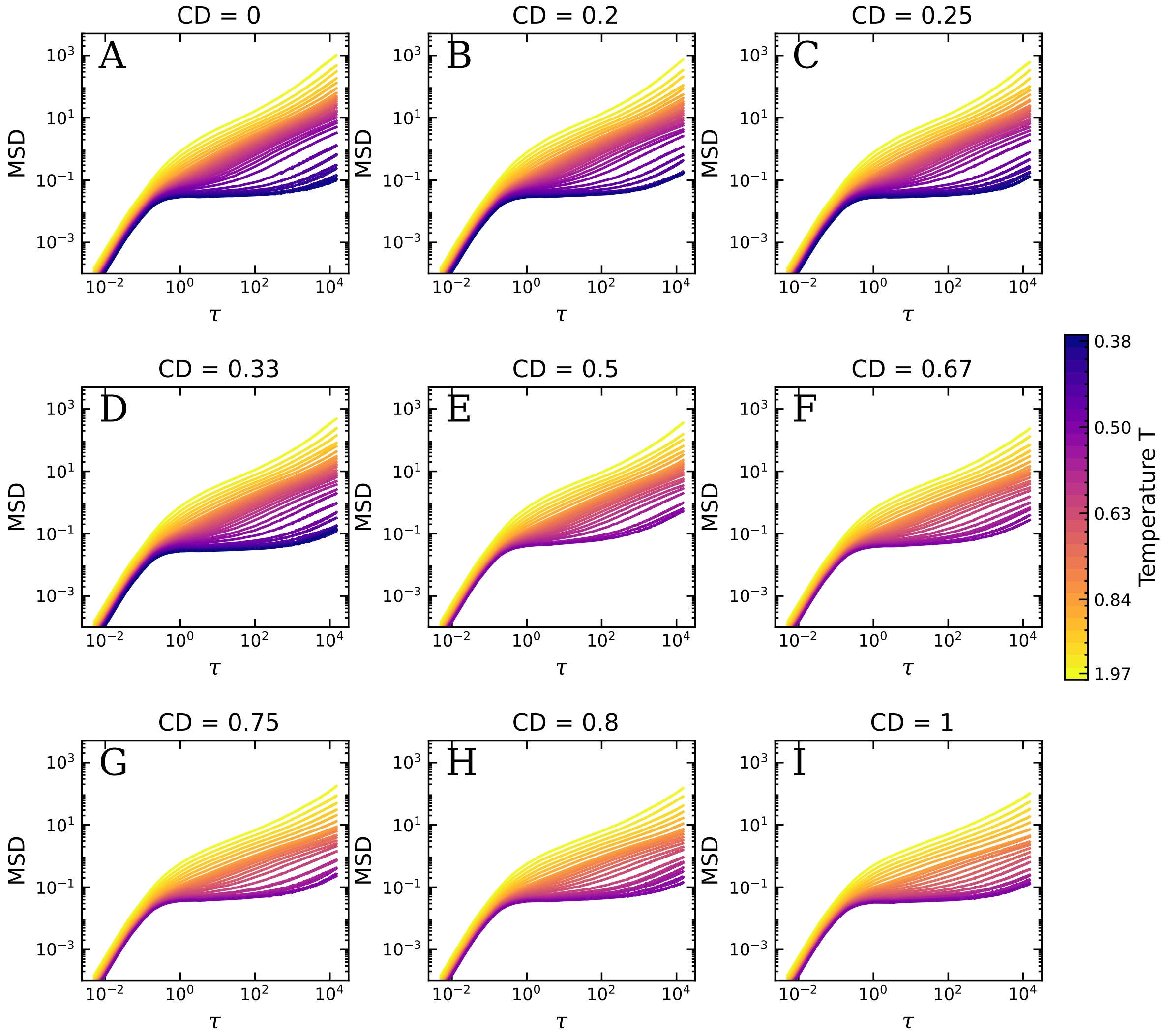


**Fig. S8**: The mean squared displacement ($MSD$) of compleximers with varying charge density ($CD$) at a side chain length $SC = 1$. **(A)**, $CD = 0$. **(B)**, $CD = 0.2$. **(C)**, $CD = 0.25$. **(D)**, $CD = 0.33$. **(E)**, $CD = 0.5$. **(F)**, $CD = 0.67$. **(G)**, $CD = 0.75$. **(H)**, $CD = 0.8$. **(I)**, $CD = 1$.

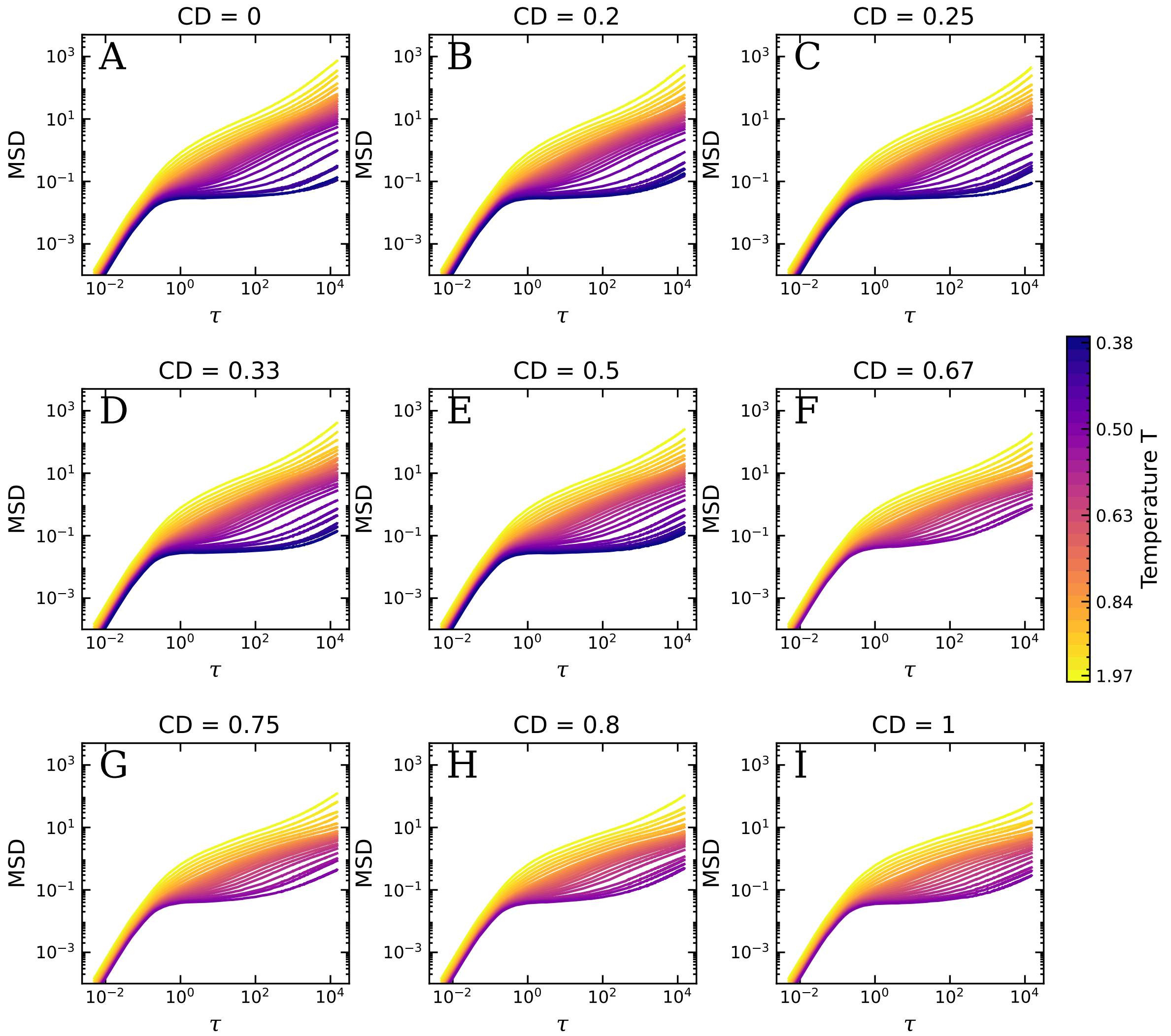


**Fig. S9**: The mean squared displacement ($MSD$) of compleximers with varying charge density ($CD$) at a side chain length $SC = 2$. **(A)**, $CD = 0$. **(B)**, $CD = 0.2$. **(C)**, $CD = 0.25$. **(D)**, $CD = 0.33$. **(E)**, $CD = 0.5$. **(F)**, $CD = 0.67$. **(G)**, $CD = 0.75$. **(H)**, $CD = 0.8$. **(I)**, $CD = 1$.

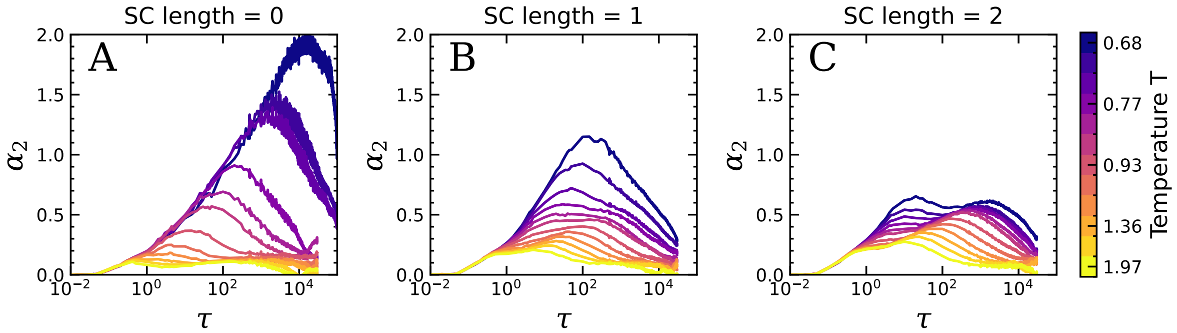


**Fig. S10**: The non-Gaussian parameter ($\alpha_2$) of compleximers with varying side chain length ($SC$) at a charge density $CD = 1$. **(A)**, $SC = 0$, **(B)**, $SC = 1$, **(C)**, $SC = 2$, **(D)**, $SC = 3$.

The slowing down of the particles upon approaching the glass transition is often accompanied by dynamic heterogeneities, where the particle mobility is different in different locations of the material. To characterize these dynamic heterogeneities, we calculate the non-Gaussian Parameter ($\alpha_2$), defined as:

$$\alpha_2(t) = \frac{3\langle \Delta r^4(t) \rangle}{5\langle \Delta r^2(t) \rangle^2} - 1,$$

where $\langle \Delta r^2(t) \rangle$ is the mean squared displacement ($MSD$) and $\langle \Delta r^4(t) \rangle$ is the fourth moment of the displacement distribution. A value of $\alpha_2(t)$ close to zero indicates a Gaussian displacement distribution, while a non-zero value reflects non-Gaussian dynamics, typically arising in systems exhibiting spatial heterogeneity of the dynamics. We find that $\alpha_2$ peaks around the cage relaxation time, which is the time scale of maximum cooperative rearrangements, typically corresponding to $\tau_\alpha$. As the temperature decreases the peak shifts to longer times and becomes higher, indicating more pronounced dynamic heterogeneities . We find similar behavior across systems with varying $SC$ length and varying charge density.

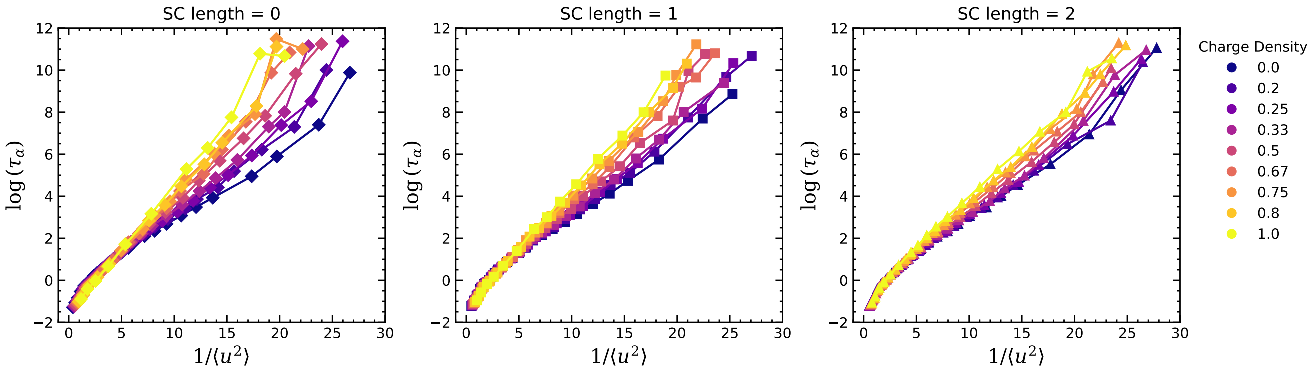


**Fig. S11**: $\log(\tau_\alpha)$ vs $1/\langle u^2 \rangle$ for different $SC$ and $CD$.
